# The role of extracellular vesicles in cholangiocarcinoma

**DOI:** 10.1186/s12935-020-01526-y

**Published:** 2020-09-04

**Authors:** Mingzhen Bai, Wenkang Fu, Gang Su, Jie Cao, Long Gao, Chongfei Huang, Haidong Ma, Jinduo Zhang, Ping Yue, Bing Bai, Yanyan Lin, Wenbo Meng, Xun Li

**Affiliations:** 1 The First Clinical Medical College, Lanzhou University, Lanzhou, 730000 China; 2 Department of Special Minimally Invasive Surgery, The First hospital of Lanzhou University, Lanzhou, 730000 China; 3 Division of Scientific Research and Development Planning, The First Hospital of Lanzhou University, Lanzhou, 730000 China; 4 Institute of Genetics, School of Basic Medical Sciences, Lanzhou University, 730000 Lanzhou, China; 5 Gansu Province Institute of Hepatopancreatobiliary, 730000 Lanzhou, China; 6 Gansu Province Key Laboratory Biotherapy and Regenerative Medicine, 730000 Lanzhou, China; 7 The Second Department of General Surgery, The First Hospital of Lanzhou University, 730000 Lanzhou, China

**Keywords:** Cholangiocarcinoma, Extracellular vesicles, Exosomes, Biomarkers, Therapy

## Abstract

Cholangiocarcinoma (CCA) is a rare tumor that arises from cholangiocytes, the epithelial cells of the bile duct. The tumor is characterized by insidious onset, high degree of malignancy, poor prognosis and high recurrence rate. Due to the lack of specific biomarkers, it is difficult to diagnose CCA early and evaluate prognosis. Extracellular vesicles (EVs), which include apoptotic bodies, microvesicles and exosomes, have emerged as having important roles in cell-to-cell communication in both normal physiology and pathological conditions. Some research has found that EVs play a crucial role in the occurrence and development of CCA. EVs can carry specific molecular substances such as nucleic acids and proteins, which have potential for the diagnosis and therapy of CCA. This article reviews the current knowledge on the role of EVs in CCA. We highlight EVs and their functions in the physiology and pathophysiology of CCA, and discuss their therapeutic potential and their role as biomarkers.

## Introduction

Cholangiocarcinoma (CCA) is the most common malignancy of the biliary tree, accounting for approximately 3% of all gastrointestinal tumors and is the second most common primary liver tumor after hepatocellular carcinoma (HCC) [1, 2]. The incidence of CCA varies geographically and demographically, and the overall incidence is still on the rise worldwide [3]. Although the 1-year survival has improved over time, the 5-year survival is less than 10% [4]. Most patients with CCA do not possess exact risk factors and the clinical manifestation may be nonspecific, even in the late-stage of the disease [5]. As such, early detection could improve survival, and this highlights the requirements for novel methods to diagnose and treat CCA.

Recently, the emerging role of extracellular vesicles (EVs) in cholangiocarcinoma progression has attracted extensive attention. To date, the role of EVs has changed from being nonfunctional discards of cellular components to the current research focus [6, 7]. EVs are nanosized, membrane-bound vesicles released from cells that can transport cargo, including DNA, RNA, and proteins, between cells as a form of intercellular communication [8, 9]. With so many contents, the nascent field of EVs has evolved to have a sharper focus, especially in oncology [10]. Therefore, EVs and their derived cargos have emerged as new biomarkers for tumor diagnosis. Tumor-derived EVs play a key role in modulating intercellular communication between tumor and stromal cells in local and distant microenvironments [11, 12]. In addition, EVs can still be used for therapeutic purposes as targets, immunomodulators and delivery vehicles [13]. In this review, we highlight and discuss about the relationship between EVs and CCA, with a special focus on their roles and potential clinical application values as biomarkers and therapeutic targets in CCA.

## Epidemiology, risk factors, diagnosis and treatment of CCA

Cholangiocarcinoma is a devastating tumor, currently classified as intrahepatic (iCCA), perihilar (pCCA), or distal (dCCA), characterized by varying degrees of desmoplastic reaction and increasing morbidity and mortality worldwide [14, 15]. While it is more common in Asia, its incidence has risen significantly in Europe and North America in recent decades [16]. Approximately 35,660 patients with iCCA are diagnosed each year in the United States, and the 5-year survival rate is about 10% [17, 18]. The incidence of CCA is highest in Thailand, with 113 per 100,000 men and 50 per 100,000 women per year [19]. In most case of CCA, the etiology is unknown, but chronic inflammation and cell injury in the bile duct shown a high risk of occurrence of CCA [14]. Pathologically, the release of inflammatory cytokines, increased cell death and proliferation, as well as changes in the liver in fibrosis contribute to the occurrence of tumor [20]. Primary sclerosing cholangitis is considered as the main risk factor for CCA [21]. The diagnostic basis mainly includes imaging methods (ultrasound, computed tomography, magnetic resonance imaging and fluorodeoxyglucose positron emission tomography), histological analysis of a tumor biopsy and serum nonspecific tumor biomarkers, such as carbohydrate antigen 19-9 (CA19-9) and carcinoembryonic antigen (CEA) [22–24]. However, the sensitivity of these tests in the diagnosis of CCA is limited, especially in the early stages of the disease [25]. Management strategies include multispecialty treatments, with consideration of surgical resection, targeted radiation therapy, and systemic chemotherapy [24, 26, 27]. Surgical resection is the only potentially curative treatment, but the majority of patients present with advanced cancer and recurrence after resection is common [16]. Lymph node metastasis is a prominent feature of CCA [28]. Diagnosed CCA is usually advanced and often inoperable, leading to a poor prognosis [29].

## Details about EVs

### Classification of EVs

According to the current knowledge of their biogenesis, EVs can be broadly divided into two main categories: microvesicles and exosomes [30, 31]. Microvesicles (MVs, 100–1000 nm in diameter) are secreted by the shedding or outward budding of the plasma membrane [32]. The formation of microvesicles is the result of the dynamic interaction of phospholipid redistribution and cytoskeletal protein contraction [33]. Since little information about biogenesis and MV release is understood, we focused on the function of exosomes in CCA.

### Characteristics of exosomes

In 1983, Johnstone et al. first isolated exosomes from the supernatant of sheep reticulocyte culture medium [34]. However, due to the role of exosomes being unclear at that time, it is thought to be a nonfunctional particle in the cell. Whereas exosomes are 40–150 nm, endosome-derived, small EVs which are secreted by most cells [35]. RNA, DNA and proteins are reported to be actively and selectively incorporated into intraluminal vesicles, which reside within multivesicular endosomes and are the precursor of exosomes [36, 37]. The release of exosomes into the extracellular space is facilitated by the fusion of multivesicular bodies (MVBs) limiting the membrane with the plasma membrane [35, 38]. Then, exosomes can be carried away by extracellular fluid, such as saliva, urine, blood, semen, amniotic fluid, ascites, alveolar lavage fluid, milk, synovial fluid and cerebrospinal fluid, and taken up by other cells [39–41]. Besides, bile included [42]. The function of exosomes depends on the type and contents of their parent cells. Exosomes from normal cells play a role in maintaining stability in vivo, while tumor cell-derived exosomes are associated with tumor progression [40, 43].

In addition, we need to identify exosomes that can distinguish them from other EVs by their size and proteins markers. For instance, MVB-associated proteins (tumor susceptibility gene 101 (TSG101) and ALIX) [44, 45], fusion proteins, membrane transport proteins (flotillin-1) [46], tetraspanins (CD63, CD81and CD9) [36], and heat shock proteins (HSP70.1 and HSP20) [42, 47] are often used as protein markers to recognize exosomes in scientific research. Currently, with the identification of a large number of cargo molecules in exosomes, their functions, including regulating immune function, enhancing metastasis, and modulating intercellular communication, have also been explored in tumor cells [48–50]. Some research shows that exosomes from highly metastatic breast cancer cells (4T1 and EO771 cells) can modulate the favorable microenvironment for lung and liver metastasis colonization [51]. In addition, various immune cell-derived exosomes, such as natural killer cells, dendritic cells, macrophages, neutrophils, mast cells and myeloid-derived suppressors, can act on tumor cells, modulating the growth, metastasis and response to chemotherapy [48, 52]. Next, we mainly elucidate the role of exosomes in CCA.

## Characterization of EVs associated with CCA

As mentioned above, EVs can be produced in both cells and body fluids, and their characteristics are similar to each other. Although EVs can be extracted by many methods, differential centrifugation is the most widely used method. Accumulated evidence has reported that EVs can be extracted from the serum, bile and CCA cells of patients with bile duct carcinoma and identified by specific markers, as shown in Table 1. The morphological and molecular characteristics of these EVs indicate that they are mainly exosomes. However, the contents of EVs are numerous, and their functions are different, and these need to be further studied.

**Table 1:** Characterization of EVs in bile, serum, cholangiocytes and CCA cells

The source of EVs	(TEM)/ (NTA)	Biomarkers	References
CCA bile	Rounded, cup-shaped, double-membrane-bound vesicles, 50–750 nm in diameter	TSG101, ALIX, CD9, HSP70.1	[42]
	Spherical structures of vesicles,30–110 nm in diameter, the mode of exosomes sizes is 72.2 nm	CD63, CD81	[99]
	Spherical structures, the mode of EVs sizes is 84 nm, 30–110 nm in diameter, 3 × 10^11^ EVs/ml bile	TSG101, CD63	[98]
CCA serum	Round morphology, ~ 180 nm in diameter	CD9, CD63, CD81	[104]
	50–750 nm in diameter	TSG101, ALIX, CD9, HSP70.1	[42]
KKU-M213 KKU-100	Crescent shaped membrane invagination, 40–100 nm in diameter	Flotillin-1, TSG101, CD81, CD63	[64]
	50–150 nm in diameter	Flotillin-1, CD81, TSG101	[73]
KMBC	Spherical structures of vesicles, a mean size of 137 ± 960 nm	ALIX, CD9, CD81	[61]
KKU-M213D5	40–100 nm in diameter	ALIX, TSG101	[105]
RBE	30–120 nm in diameter	ALIX, TSG101, CD63	[65]
HuCCA-1	50–150 nm in diameter	Flotillin-1, CD81, TSG101	[73]
**H69**	50–150 nm in diameter	Flotillin-1, CD81, TSG101	[73]

CCA cell lines: KKU-M213, KKU-100, KKU-M213D5, KMBC, RBE and HuCCA-1. Normal human cholangiocyte cell line: H69

*TSG101* tumor susceptibility gene 101,* TEM* transmission electron microscopy,* NTA* nanoparticle tracking analysis

## EVs regulate the progression of CCA

**Fig. 1:**
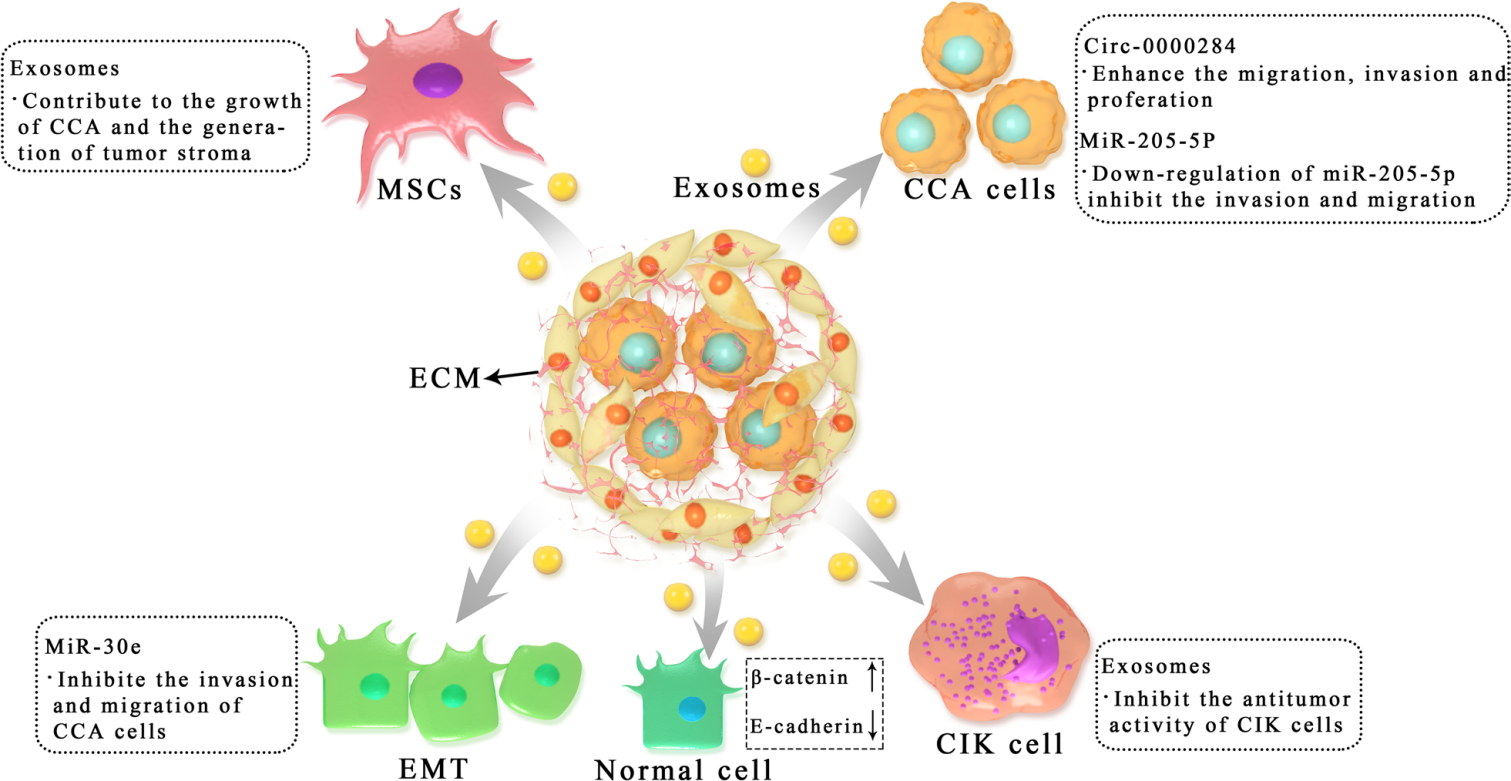
Roles of tumor cells derived exosomes in the progression of CCA. Exosomes are critically involved in CCA progression including tumorigenesis, development, immune escape and metastasis by transferring functional biomolecules. CCA cell derived exosomes induced the expression of β-catenin and decreased the expression of E-cadherin to increase motility of normal cells. CCA cell derived exosomes interact with marrow mesenchymal stem cells (MSCs) to modulate the microenvironment and promote CCA growth. Moreover, CCA cell derived exosomes participate in immune escape by inhibiting the cytokine-induced killer (CIK) cells

With the further development of EVs, increasing evidence has been presented to demonstrate the role of EVs in the progression of CCA (Fig. 1). Bile duct carcinoma usually has a dense stroma that contains immune cells (including neutrophils, tumor associated macrophages, natural killer cells, and T and B lymphocytes) and an extracellular matrix that promotes connective tissue proliferation [53, 54]. In particular, cancer-associated fibroblasts (CAFs), which communicate not only with tumor cells, but also with stromal cells, play a central role in the progression of CCA [55, 56]. CAFs influence the behavior of CCA by releasing various metabolites and soluble factors, such as vascular endothelial growth factor (VEGF)-A and VEGF-C, which may lead to dilation of the lymphatic vasculature and tumor cell intravasation [57]. Therefore, it also has the property of easy transfer [18]. In this process, EVs play a crucial role in facilitating the communication of various signals (Table 2).

**Table 2:** Summarize the function of EVs contents in CCA

EVs contents	Source	Downstream target or recipient cells	Function	References
DAMPs	Actived cholangiocytes	Macrophage	Upregulate proinflammatory cytokines and profibrogenic factors	[59]
Exosomes	HUCCT1 and KMBC	MSCs	Contribute to tumor cell growth and stromal development	[61]
Exosomes	KKU-M213	H69 cell	Induce the expression of β-catenin and reduce the expression of E-cadherin	[64]
Exosomes	RBE	CIK cell	Inhibit the antitumor activity of CIK cells	[65]
MiR-205-5p	KKU-M213	CCA cell	Down-regulation of miR-205-5p can inhibit invasion and migration of CCA cells	[73]
MiR-195	LX2	A rat model of CCA	Inhibit CCA growth and improve survival in a rat model of CCA	[77]
MiR-30e	HuCCT1	CCA cell	Inhibit CCA cell invasion and migration via inhibit EMT	[81]
Circ-0000284	HuCCT1 and RBE	Gene LY6E	Enhancing the migration, invasion and proliferation of CCA cells	[86]

CCA cell lines: KKU-M213, KMBC, RBE and HuCCT-1. Normal human cholangiocyte cell line: H69. A human liver stellate cell line: LX2

### The function of EVs in tumor microenvironment

Cholangiocytes can be actively involved in the development of bile duct disease by stimulating the recruitment and activation of inflammatory cells in the bile duct microenvironment [58]. Katsumi et al. found that activated bile duct cells are involved in activating the proinflammatory polarization of damage-associated molecular patterns (DAMPs) through the receptor for advanced glycation end products (RAGE) signaling pathway by releasing DAMPs as EV cargo [59]. Prior to this, Masyyuk et al. verified exosomes in rat bile [60]. Meanwhile, in the process of studying the cilium interaction of bile duct cells, exosomes were found to induce intracellular signals and functional responses, verifying that bile exosomes participate in intercellular signal communication [60]. This laid a foundation for the influence of bile duct carcinoma exosomes on tumor progression.

In the study of the tumor microenvironment of CCA, Haga et al. exposed marrow mesenchymal stem cells (MSCs) to CCA cell-derived EVs, which enhanced expression of alpha-smooth muscle actin mRNA and release of cytokines/chemokines such as IL-6, thus regulating the tumor microenvironment and promoting the growth of CCA. In addition, CCA cell-derived EVs can contribute to the formation of the tumor stroma through the fibroblast differentiation of MSCs [61]. This further revealed the effect of tumor cell-derived EVs on the local microenvironment. However, they did not identify the specific contents of the EVs. Moreover, proteins in exosomes are essential agents for tumor growth [62, 63]. Another study demonstrated the protein spectrum of CCA-derived exosomes and their potential roles. These researchers isolated exosomes from CCA cell lines (KKU-M213 and KKU-100) and incubated them with normal bile duct cells (H69). After proteomics analysis, exosomes were found to be internalized into H69 cells, resulting in migration and invasion of H69 cells, but failing to induce proliferation. In addition, the exosomes of KKU-M213 cells induced the expression of β-catenin and decreased the expression of E-cadherin, suggesting that exosomes might induce the migration and invasion of bile duct cells through the direct transfer of oncogene proteins between cells, thus affecting the specific intracellular mechanism related to CCA carcinogenesis [64]. The proteomics analysis of normal bile duct cells and CCA cells confirmed the differences between these two cell types, and these differences need to be further studied. Moreover, exosomes from another CCA cell line (RBE) could inhibit the antitumor activity of cytokine-induced killer (CIK) cells by downregulating the populations of CD3^+^, CD8^+^, NK (CD56^+^) and CD3^+^CD56^+^ cells and secreting TNF-α and perforin [65]. According to some research, mutations in the IDH1 gene is common in a variety of tumors, including iCCA [66, 67]. Recent studies have shown that the R132C mutation is the most common type of IDH1 mutation in iCCA. The IDH1^R132C^ mutation leads to the downregulation of P2RX7 expression, which further affects the exosome secretion of tumor cells, ultimately affecting the progression of CCA [68]. However, more functional experiments on P2RX7 are needed.

### Exosomal noncoding RNAs in CCA

In addition, noncoding RNA also play a crucial role in tumor development [69]. As a functional regulatory molecule, noncoding RNAs, such as microRNAs (miRNAs) and circular RNAs (circRNAs), could mediate cellular processes, including chromatin, transcription, posttranscriptional modification and signal transduction, and, of course, predict prognosis [69–71].

#### Exosomal microRNAs in CCA

MiRNAs are small noncoding RNAs composed of 19–24 nucleotides [72]. Kitdumrongthum et al. found different miRNA expression profiles in the exosomes released by CCA cells and cholangiocytes, and many miRNAs with abnormal regulation had functions related to a variety of oncogenes [73]. For example, miR-205-5p, the most upregulated miRNA, down-regulation of miR-205-5p can inhibit invasion and migration of CCA cells [73]. Moreover, miR-205-5p has also been reported in other cancers, such as breast cancer and gastric cancer [74, 75]. However, in contrast to CCA, the expression of miR-205-5p is downregulated in breast cancer, and miR-205-5p has an antitumor effect in breast cancer [76]. Li and colleagues reported that EVs could transport miRNA species between human CCA cells and CAFs. They used LX2-derived EVs carrying miR-195 to inhibit CCA growth and improve survival in a mouse model of CCA, demonstrating the communication between the tumor and microenvironment [77]. Epithelial–mesenchymal transition (EMT) is a biological process in which epithelial cells gradually change and lose epithelial characteristics and differentiate into mesenchymal phenotypes, and it is closely related to the invasiveness and motility of tumor cells [78–80]. CCA-derived EVs could transfer miR-30e and inhibit EMT by directly targeting the Snail in receptor cells, thus inhibiting the invasion and migration of bile duct cancer cells [81]. In addition, miR-200a/c-3p in serum exosomes was significantly positively correlated with the CCA stage, and mainly involved in lymphatic metastasis of tumors [82].

#### **Exosomal circRNAs in CCA**

Recent research has demonstrated that circRNAs also have a biological role in CCA [83, 84]. Wang et al. found that the level of circ-0000284 was increased in CCA cell lines, tumor tissues and plasma exosomes, thus enhancing the migration, invasion and proliferation of CCA cells in vivo and in vitro. The circ-0000284/miR-637/LY6E regulatory axis was involved in this process [85, 86]. In addition, exosome-mediated circ-0000284 could stimulate the malignant behavior of surrounding normal cells and ultimately promote the progression of CCA [86].

## EVs as novel biomarkers for CCA

**Fig. 2:**
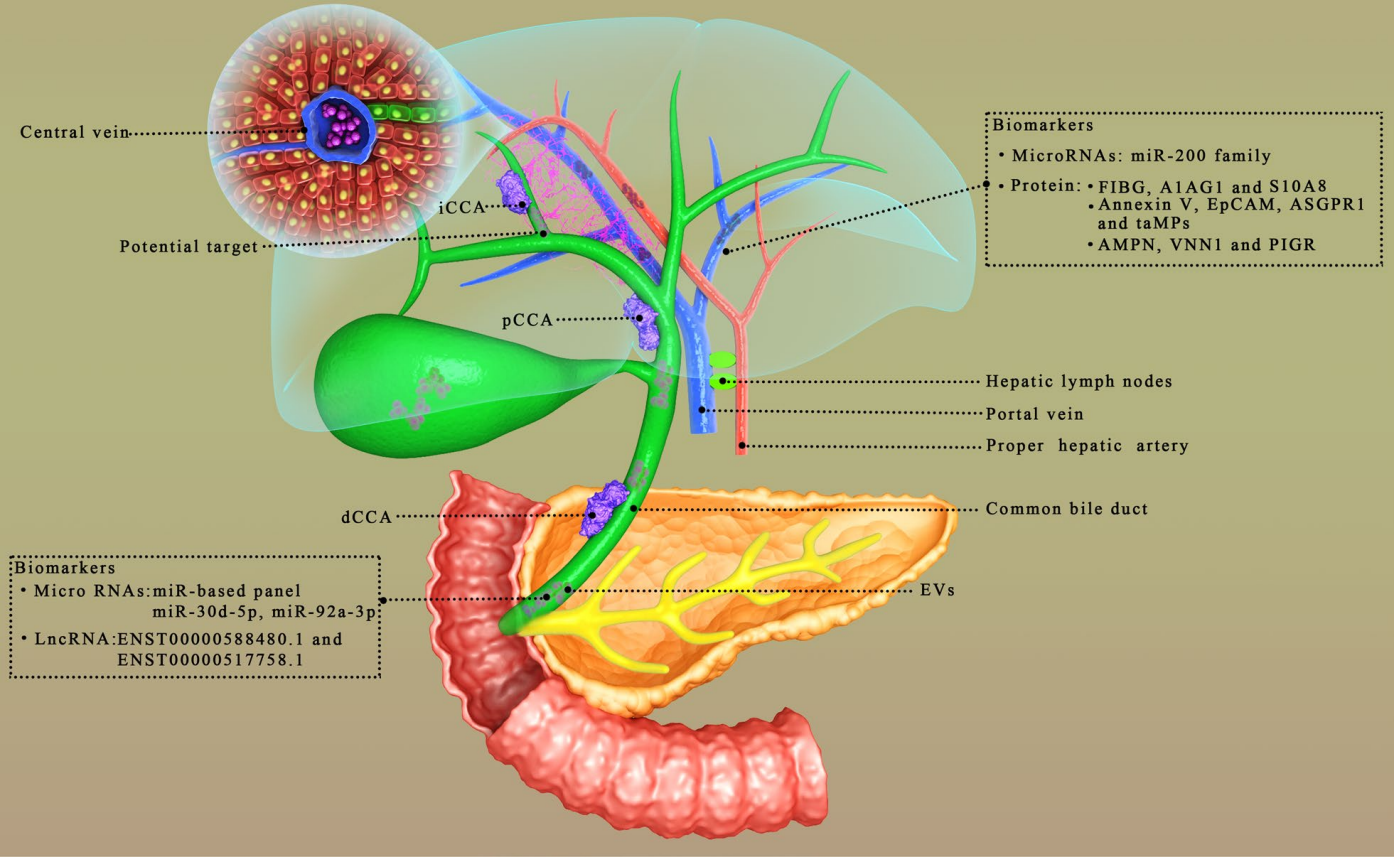
EVs can exist in bile and blood, which is helpful for disease detection and diagnosis

A major reason for the poor prognosis of CCA is the lack of early detection. Late diagnosis delays optimal treatment and leads to lower survival. Therefore, it is necessary to develop new methods for the diagnosis of CCA. In recent years, studies have shown that EVs have huge potential in the diagnosis of diseases due to their unique properties and great progress has been made in studying of EVs as tumor diagnostic markers [41, 87, 88]. EVs in bile and blood have opened up new ideas for the early diagnosis of CCA. Compared with traditional CA-199 and CEA, EVs have higher diagnostic value in CCA, which is summarized in Fig. 2.

### The isolation of EVs

**Fig. 3:**
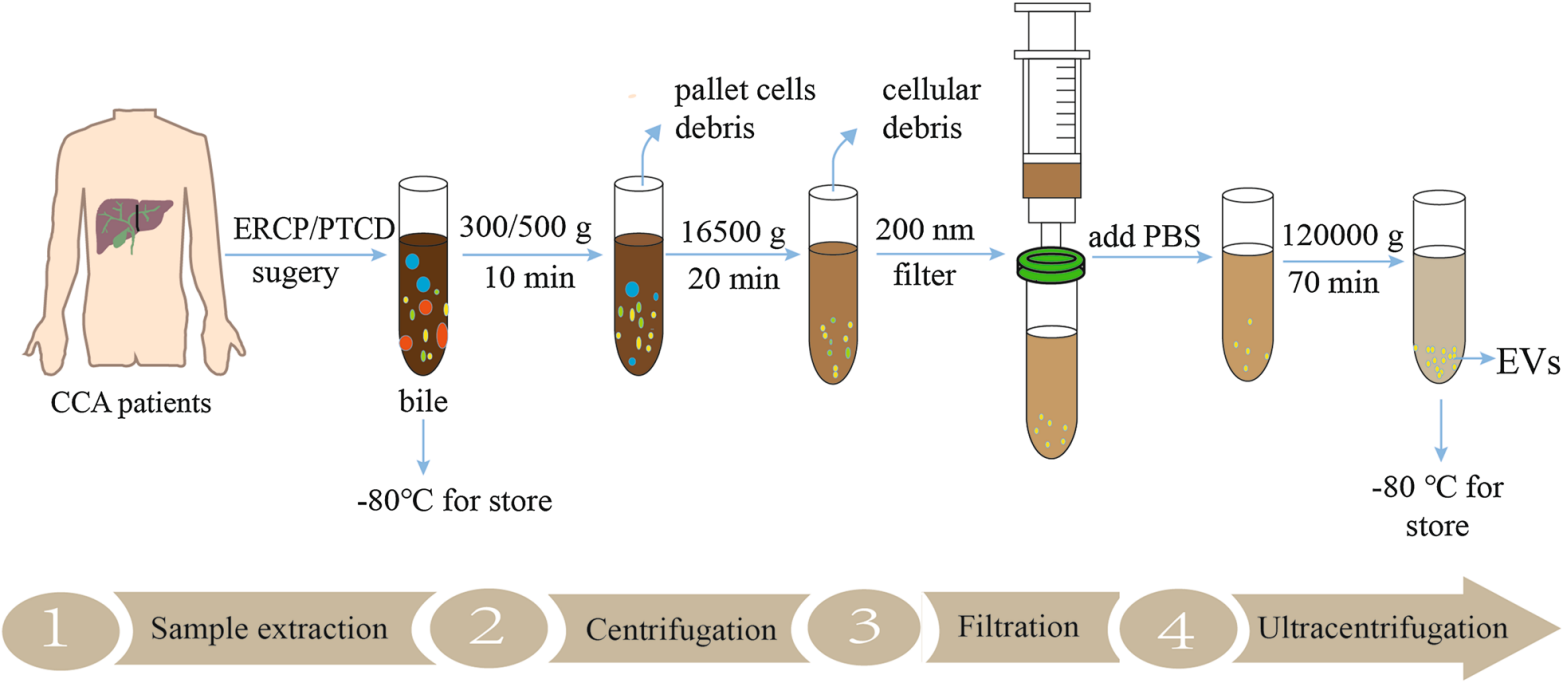
Schematic representation of the isolation of EVs from bile by ultracentrifugation

With the development of technology, many exosome separation and purification techniques, including ultracentrifugation, ultrafiltration, immunoaffinity capture, size-exclusion chromatography, microfluidic techniques and charge neutralization-based polymer precipitation, have been exploded [89–91]. Among them, the most common application is ultracentrifugation, which is the gold standard for exosome separation, even though it has some limitations, such as having a low efficiency and being time consuming [92–94]. In the CCA research process, the EVs in the culture media of CCA were separated mainly by ultracentrifugation [61, 64, 65]. Similarly, EVs are extracted from the serum by ultracentrifugation, and polymer-based precipitation kit [95, 96]. However, there is a study that supports some commercial kits, such as ExoQuick and miR-CURY, as being better than ultracentrifugation, even with a limited quantity of EVs [92]. Bile is a lipid-rich fluid, that is secreted primarily by hepatocytes, and contains almost all body components: lipids, proteins, carbohydrates, vitamins, mineral salts and trace elements [96, 97]. Because of the characteristics and complexity of bile extraction, we mainly summarize the isolation of bile here. Bile samples of CCA are usually collected by endoscopic retrograde cholangiopancreatography (ERCP), percutaneous transhepatic biliary drainage (PTBD) and surgery, and then bile is immediately centrifuged at 4 ℃ to remove the cell debris and filtered through a 200 nm filter. Finally, the supernatant is collected and added to an ultracentrifuge tube, diluted with PBS for further purification, mainly including ultracentrifugation, PEG precipitation, membrane filtration and affinity purification [98–100]. Some research, however, initially collected bile and then rapidly diluted with PBS for further centrifuge [42]. The isolation of EVs were used immediately or stored at − 80 °C until use (Fig. 3). However, research in this area is limited, and there is still much room for improvement in the separation and purification of exosomes.

### Bile EVs

MiRNA in EVs is protected the membrane from degrading enzymes, and this determines its highly stable characteristics in the extracellular environment, thus, miRNA is used for the diagnosis of a variety of cancers, including CCA [101, 102]. Li et al. identified and characterized EVs in human bile for the first time, and miRNA-laden EVs in human bile could be used for the diagnosis of CCA. They defined a new biliary vesicle miR-based panel (miR-191, miR-486-3p, miR-1274b, miR-16 and miR-484) for the diagnosis of CCA with a sensitivity of 67% and specificity of 96% [98]. This research initiates studying EVs in bile. Han and coworkers found that the expression levels of miR-30d-5p and miR-92a-3p in the bile of CCA patients were specifically upregulated compared with those in the bile of patients with benign biliary disease [103]. Compared with CEA and CA19-9, miR-30d-5p had the best effect in differentiating CCA and common bile duct disease, with a sensitivity of 81.1%, specificity of 60.5% and area under the curve (AUC) value of 0.730. In addition, the identification of CCA using the combination of the two bile miRNAs and serum CA19-9 levels was weaker than that of miR-30d-5p alone [103].

Long noncoding RNAs (lncRNAs) in bile also play the same role in CCA as miRNAs. Ge et al. conducted a series of experiments on bile exosomes, and showed that the expression of the lncRNAs ENST00000588480.1 and ENST00000517758.1 in the CCA group was significantly increased compared with that of the control group. The AUC of the combined detection of the two lncRNAs was 0.709, and the sensitivity and specificity were 82.9% and 58.9%, respectively [99]. Moreover, with the increase in the tumor TNM stage, the expression levels of ENST00000588480.1 and ENST00000517758.1 were significantly increased, and they could be potential diagnostic markers [99]. The content of EVs in bile can be used as a diagnostic marker, and the concentration of EVs also has potential diagnostic value. Severino et al. found that the accuracy of the bile EV count in the diagnosis of CCA was 100% with a threshold value of 9.46 × 10^14^ nanoparticles, with an AUC of 1, and this could correctly distinguish malignant and nonmalignant common bile duct stenoses in this research [42].

### Serum EVs

There are also some diagnostic markers of CCA in serum EVs. The new proteomic features found by Ander Arbelaiz in serum EVs of primary sclerosing cholangitis (PSC), CCA and HCC patients have potential diagnostic value [104]. For example, fibrinogen gamma chain (FIBG), alpha-1-acid glycoprotein 1 (A1AG1) and S100A8 (S10A8) proteins have the strongest differential diagnoses of CCA and PSC, with AUC values of 0.796, 0.794 and 0.759, respectively [104]. Similarly, using proteomic methods, Weeraphan et al. studied the exosomal phosphoproteome of M213 and M213D5 in CCA cells, and showed that Ser255 of HSP90B was highly phosphorylated in tissues of CCA patients with a low TNM stage (I and II) compared to those with a TNM stage of III or IV. ROC analysis showed that HSP90B-S255 was a new potential biomarker for metastatic CCA with an AUC value of 0.936 (sensitivity 87.27%, specificity 97.62%) [105]. Moreover, Shen et al. studied exosomal miRNA in peripheral blood samples from CCA patients and healthy controls. The results showed that the serum extracellular miR-200 family, especially miR-200c-3p, had the strongest diagnostic ability for CCA than that in serum CA19-9, with an AUC of 0.93, which is worth further study [82]. Meanwhile, positive of Annexin V, epithelial cell adhesion molecule (EpCAM), asialoglycoprotein receptor 1 (ASGPR1) and tumor-associated microparticles found in serum EVs were shown to have the potential to differentiate HCC and CCA from tumor-free individuals; after tumor resection, the number of these microparticles decreased, which proved a correlation with the presence of the tumor [95].

### Summary of the role of EVs in diagnosing CCA

The studies above indicate that miRNAs, lncRNAs and proteins in blood and bile could be used as diagnostic indicators of CCA (summarized in Table 3). It can be concluded that although there are many substances in EVs, exosomes are the main diagnostic agents. Exosomes coated with lipid bilayers are more stable and more suitable as diagnostic markers [8]. The concentration of EVs in bile may increase as a result of bile flow disorder caused by bile duct stenosis or obstruction in CCA patients [106]. In addition, potential tumor-derived biomarkers may be secreted directly into bile by adjacent CCA cells, and local sampling may be more likely to detect candidate biomarkers directly related to the tumor [106]. Therefore, compared with circulating serum sampling, bile sampling can improve diagnostic performance [103]. However, the study of the EVs in CCA is still not complete. For example, the mechanism of EVs in bile duct cancer is not clear and how to efficiently extract and detect EVs in body fluids remains unclear.

**Table 3:** EVs as diagnostic biomarkers for CCA

Biomarkers	AUC	Sensitivity	Specificity	Reference
CCA vs. PSC BBO				[98]
Bile: miR-based panel	Null	67%	96%	
CCA vs. BBD				[103]
Bile: miR-30d-5p	0.730	81.1	60.5	
miR-92a-3p	0.652	65.7	66.7	
Serum: CA19-9	0.675	70.3	64.6	
CEA	0.603	64.9	60.4	
CCA vs. BBO				[99]
Bile lncRNAs: ENST00000588480.1 and ENST00000517758.1	0.709	82.9	58.9	
Serum: CA19-9	0.729	74.3	71.4	
CCA: TNMs I, II vs. TNMs III, IV				[105]
Serum: HSP90B-s255	0.936	87.27	97.62	
MCBDS vs. NMCBDS				[42]
Concentration of EVs	1.000	Null	Null	
Serum: CA19-9	0.733	Null	Null	
CCA vs. HC				[82]
Serum: miR-200c-3p	0.930	Null	Null	
Serum: CA19-9	0.780	Null	Null	
CCA vs. PSC				[104]
FIBG	0.796	Null	Null	
A1AG1	0.794	Null	Null	
S10A8	0.759	Null	Null	
Serum: CA19-9	0.819	Null	Null	
CCA vs. HC				
AMPN	0.878	Null	Null	
VNN1	0.876	Null	Null	
PIGR	0.844	Null	Null	
Serum: CA19-9	0.907	Null	Null	

## Application of EVs in CCA therapy

To date, due to the difficulty in the early diagnosis of CCA, there are few treatment options, and radical surgical resection is the only effective treatment method [107, 108]. Postoperative adjuvant chemotherapy can improve the survival and cure rates, but the effect of chemotherapy is not enough [18]. With the development of research on EVs, the treatment of CCA has a new direction. The characteristics of tumor-secreted EVs in regulating the immune microenvironment illustrate their clinical potential in immunotherapy, therapeutic targeting and drug delivery [11, 109].

### Therapeutic targets

Since the tumor microenvironment promotes the progression and invasion of CCA, targeting the microenvironment and related cells is a strategy for the treatment of CCA [110, 111]. Chen et al. found that RBE-derived exosomes could inhibit the antitumor activity of CIK cells [65]. This suggests that the effect of CIK cell-based immunotherapy is related to EVs, which may be a potential therapeutic target. In addition, circ-0000284 may be a therapeutic target for CCA. Wang et al. proved that the knockdown of exosomal circ-0000284 inhibited CCA growth and metastasis in vivo through animal experiments [86]. Besides, according to Zhang’s research, mutations in the IDH1 gene alter the function of IDH1 and affect the development of CCA by promoting exosome release [68]. Interestingly, IDH1 inhibitors have been reported. For example, the safety and clinical efficacy of mutant IDH1 inhibitor ivosidenib in the recurrence or refractory IDH1-mutated acute myeloid leukemia were demonstrated [112]. At present, inhibitors of IDH1 (AG120 and IDH305) are being tested in iCCA patients [113]. This provides a potential treatment of CCA. Furthermore, one study reported that inhibition of miR-205-5p in the exosomes of CCA could reduce the invasion and migration of CCA, and the miR-200 family was associated with drug resistance [73, 114].

### Drug delivery

EVs are natural membrane vesicles involved in intercellular communication; accumulating evidence has revealed that EVs have the characteristics of stability and low immune reactivity, and exosomes in EVs can effectively transport a variety of different types of cargo to target cells [109, 115]. As a result, EVs can also be selected as a therapeutic tool to modulate the function of CCA cells by delivering cargo media [35, 116]. Stromal derived EVs are suitable for the delivery of materials to CCA cells, and this property can be exploited for delivering antitumor therapy to CCA cells [117]. Li et al. proved that EVs could transport miR-195 from fibroblasts to cancer cells. In addition, fibroblasts-derived EVs loaded with miR-195, play a key role in the CCA rat model, by reducing the size of tumors and improving survival in the treated rats [77]. Moreover, after incubating the miR-30e-enriched EVs with CCA cells, Zhang found that the expression of miR-30e in receptor CCA cells increased, which ultimately regulated the invasion and migration of cells. This demonstrates that miR-30e-enriched EVs can be ingested by recipient cells as a means of transferring miR-30e [81]. A recent study showed that methotrexate-loaded tumor-cell-derived microvesicles were injected into the bile duct lumen of patients with extrahepatic CCA, which mobilized and activated neutrophils and alleviated biliary obstruction [118]. These studies suggest that EVs carrying designed cargo can be used as media carriers to manage the progression of CCA.

## Conclusion and future prospects

EVs are present through the occurrence, development and metastasis of tumors, providing new clues for the diagnosis and treatment of CCA. Many substances in EVs, such as miRNAs, circRNAs, proteins and even EV concentrations, can be used as new biomarkers. It is also important to improve the early diagnosis of CCA. Moreover, according to the role of EVs in the tumor microenvironment and immunity, corresponding targeted drugs and immunotherapy can be used for the treatment of CCA. Exosomal programmed-death ligand (PD-L1) has been found to contribute to immunosuppression and has been associated with the anti-PD-1 response, although it is mainly involved in melanoma [119]. Moreover, PD-L1expression in iCCA and perihilar cholangiocarcinoma has also been reported, and is mainly expressed in tumors with a high density of tumor-infiltrating lymphocytes [120]. Immunotherapy for PD-L1 in CCA has entered clinical research, but the efficacy is not clear [121]. EVs have shown great potential in the immunotherapy of tumors, although there is not much data on the immunotherapy of EVs in CCA and therefore, further research is needed. In summary, this review focuses on the current research status of EVs in CCA. First, the characteristics of EVs in CCA were described. Second, the mechanism of EVs in tumor growth and metastasis was discussed. Finally, we demonstrated that the contents of EVs could be used for the clinical diagnosis and treatment of CCA. Although the existing studies have partially uncovered the mechanism of EVs in CCA, there are still a few challenging problems to solve. Firstly, the detailed mechanism to describe the role of EVs in CCA needs further clarification. In addition, standardized methods for the separation, purification and analysis of EVs in body fluids are needed. Last but not least, most of the pathophysiological studies are conducted through in vitro analysis, and there are few in vivo experiments based on EVs in animal models. Follow-up studies should be conducted to better apply EVs to the clinical diagnosis and treatment of CCA in the future. Therefore, more efforts are needed to study the role of EVs in CCA.

## Data Availability

The materials supporting the conclusions of this review are included in the article.

## Abbreviations

CCA: Cholangiocarcinoma
HCC: Hepatocellular carcinoma
EVs: Extracellular vesicles
MVs: Microvesicles
MVBs: Multivesicular bodies
TSG101: Tumor susceptibility gene 101
HSP70.1: Heat shock proteins 70.1
HSP20: Heat shock proteins 20
TEM: Transmission electron microscopy
NTA: Nanoparticle tracking analysis
CAFs: Cancer-associated fibroblasts
VEGF-A: Vascular endothelial growth factor-A
VEGF-C: Vascular endothelial growth factor-C
RAGE: Receptor for advanced glycation end products
DAMPs: Damage-associated molecular patterns
MSCs: Marrow mesenchymal stem cells
iCCA: Intrahepatic cholangiocarcinoma
miRNAs: MicroRNAs
circRNAs: Circular RNAs
EMT: Epithelial–mesenchymal transition
CA19-9: Carbohydrate antigen 19-9
CEA: Carcinoembryonic antigen
pCCA: Perihilar cholangiocarcinoma
dCCA: Distal cholangiocarcinoma
FIBG: Fibrinogen gamma chain
A1AG1: Alpha-1-acid glycoprotein 1
S10A8: S100A8
AMPN: Aminopeptidase N
VNN1: Pantetheinase
PIGR: Polymeric immunoglobulin receptor
ERCP: Endoscopic retrograde cholangiopancreatography
PTBD: Percutaneous transhepatic biliary drainage
AUC: Area under the curve
lncRNAs: Long noncoding RNAs
PSC: Primary sclerosing cholangitis
EpCAM: Epithelial cell adhesion molecule
ASGPR1: Asialoglycoprotein receptor 1
BBO: Benign biliary obstruction
BBD: Benign biliary disease
MCBDS: Malignant common bile duct stenoses
NMCBDS: Nonmalignant common bile duct stenoses
HC: Healthy controls
PD-L1: Programmed-death ligand
